# Forecasting risk gene discovery in autism with machine learning and genome-scale data

**DOI:** 10.1038/s41598-020-61288-5

**Published:** 2020-03-12

**Authors:** Leo Brueggeman, Tanner Koomar, Jacob J. Michaelson

**Affiliations:** 1 University of Iowa, Department of Psychiatry, Iowa City, IA USA; 2 University of Iowa, Interdisciplinary Genetics Program, Iowa City, IA USA; 3 University of Iowa, Medical Scientist Training Program, Iowa City, IA USA

**Keywords:** Genome informatics, Autism spectrum disorders

## Abstract

Genetics has been one of the most powerful windows into the biology of autism spectrum disorder (ASD). It is estimated that a thousand or more genes may confer risk for ASD when functionally perturbed, however, only around 100 genes currently have sufficient evidence to be considered true “autism risk genes”. Massive genetic studies are currently underway producing data to implicate additional genes. This approach — although necessary — is costly and slow-moving, making identification of putative ASD risk genes with existing data vital. Here, we approach autism risk gene discovery as a machine learning problem, rather than a genetic association problem, by using genome-scale data as predictors to identify new genes with similar properties to established autism risk genes. This ensemble method, forecASD, integrates brain gene expression, heterogeneous network data, and previous gene-level predictors of autism association into an ensemble classifier that yields a single score indexing evidence of each gene’s involvement in the etiology of autism. We demonstrate that forecASD has substantially better performance than previous predictors of autism association in three independent trio-based sequencing studies. Studying forecASD prioritized genes, we show that forecASD is a robust indicator of a gene’s involvement in ASD etiology, with diverse applications to gene discovery, differential expression analysis, eQTL prioritization, and pathway enrichment analysis.

## Introduction

Autism Spectrum Disorder (ASD) is a heterogeneous group of developmental disorders caused by a range of genetic and environmental factors. The core diagnostic features of ASD — which manifest at a young age — are impairments in social communication and restrictive and repetitive behaviors and interests. Evidence for genetic causes of ASD is strong: monozygotic twins have near 90% concordance of ASD diagnosis^1^. Population and twin studies confirmed these findings^2^ and estimated ASD’s narrow-sense heritability to be in the 50–95% range.

While evidence of genetics’s role in autism is abundant, our understanding of the specific genes underlying the disorder’s etiology is still limited. It is estimated that a thousand or more genes may confer risk for ASD when functionally perturbed^3^. This functional perturbation can come in a number of forms, including *de novo* mutation, expression quantitative trait loci (eQTL) disruption, and inheritance of damaging rare variants. Despite these projections, the existing list of high-confidence autism risk genes — depending on the source — stands closer to the 100-gene mark^3–6^. This discrepancy is partly explained by the relatively limited number of genomic studies compared to the vast genetic heterogeneity underlying autism.

To close the gap between anticipated and known autism risk genes, several network-biology approaches were applied in the past decade. These studies leveraged large, publicly-available datasets to add context and amplify the genetic signals observed in ASD sequencing studies. These network-biology studies predicted individual genes that were often later identified as bona fide autism risk genes^7^; but they fell short of providing a useful genome-wide metric to indicate the evidence of every gene’s involvement in autism. More recently, machine learning based methods used gene interaction networks^8^, cell-specific gene expression profiles^9^, and human^10^ or cross-species^11^ brain region expression profiles to surmount this shortcoming. Importantly, these studies produced quantitative metrics that score every gene according to evidence of a role in autism. Despite these studies’ effectiveness in prioritizing autism risk genes, our preliminary investigations suggested there was still room for appreciable improvement in the classification algorithm, the training set, and the predictors. In particular, these approaches do not incorporate indicators autism involvement based on known genetic association (e.g., TADA scores) into their predictive features.

We introduce forecASD, a gene-level score indexing autism relevance that integrates prior network-biology approaches, scores of genetic association, brain gene expression, and topological information from large gene interaction networks. In three independent test sets — the recent sequencing studies MSSNG^12^, new ASC samples^6^, and the SPARK pilot data^13^ — forecASD has superior performance prioritizing *de novo* mutations, compared to other gene-level estimates of autism relevance.

In addition to prioritizing *de novo* mutations in probands, forecASD is an informative filter for a variety of genomic data types, including ASD-associated common variants and differential gene expression. Specifically, we find enrichment of ASD risk genes in downregulated ASD postmortem genes, and identify numerous biological pathways underrepresented in our current understanding of autism risk. We further demonstrate that expression quantitative trait loci (eQTL) regulating the expression of genes identified by forecASD also contain excessive association signal from the most recent genome-wide association study (GWAS) of autism. These analyses demonstrate forecASD’s generalizability beyond *de novo* mutation prioritization, as a tool that can be applied to the interpretation of rare and common variant studies, as well as gene expression studies of autism. forecASD is not a replacement for traditional approaches to genetic association, but a powerful resource for expanding our interpretative capabilities and anticipating the trajectory of gene discovery in autism.

## Methods

### Overview

**Figure 1:**
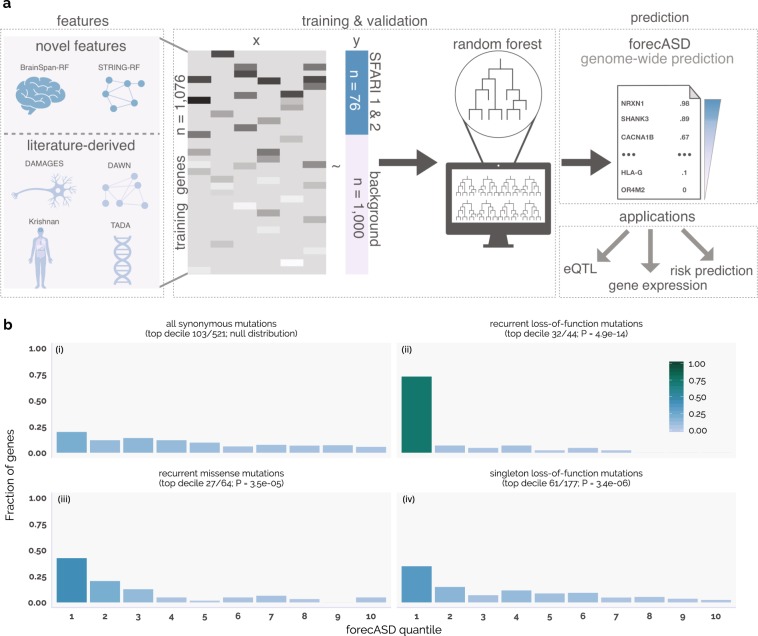
Overview and performance of forecASD. forecASD (**a**) is a random forest ensemble of features derived from the BrainSpan developmental transcriptome, the STRING network, and several previously published ASD gene prediction methods. Using SFARI 1 and 2 genes as the positive class and 1,000 background genes as the negative class, class predictions of 17,957 genes yields a ranked list with values between 0 and 1. Ranked genes are split by decile and tested for an enrichment (**b**) of multiple classes of de novo mutations derived from three independent ASD cohorts (MSSNG, SPARK pilot, ASC). The top decile synonymous mutation rate (0.196) in probands (**bi**.) is used as the expected proportion of mutations within the top decile of genes ranked by forecASD. A binomial test (fraction and p-value written below mutation type) of the enrichment of genes affected by recurrent LOF, recurrent missense, and singleton LOF mutations in the top decile showed a significant enrichment (**bii–iv**.).

forecASD utilizes stacked Random Forest ensembles, organized in two levels (shown in Supplementary Fig. S1). In the first level, two models are trained using BrainSpan^14^ gene expression and the STRING^15^ shortest paths network as features. Our training dataset consists of a positive class of high-confidence genes scored in SFARI gene^4^ as either 1 or 2 (n = 76; SFARI HC genes, retrieved January 11th 2017), and a negative class of 1,000 random genes not contained within SFARI gene. These two models produce genome-wide predictions for autism involvement. These scores are then used as features in the second level’s Random Forest model, along with genome-wide scores from previous studies (Fig. 1).

### BrainSpan, STRING, and TADA data assembly

BrainSpan data was obtained from the Allen Institute. BrainSpan is a developmental transcriptome of the human brain, made up of RNA-sequencing data covering numerous brain structures and time-points. To process this data for use in forecASD, the expression matrix from BrainSpan was downloaded and brain regions containing fewer than 20 samples were excluded (leaving 16/26 brain structures). After this initial filtering, the BrainSpan matrix consists of gene expression values from 22327 genes (rows) in 553 samples (columns), representing data from 41 unique individuals between the ages of 8 weeks post-conception to 40 years old. Despite the size of this dataset, there are not expression values for every timepoint in every region, and some region-timepoint combinations may have multiple measurements. To address this combination of sparsity and redundancy in the BrainSpan matrix, we first smoothed across available timepoints for each gene on a per-region basis (using the R^16^ stats package function lowess()), averaging out differences between redundant timepoints. Next, we linearly interpolate (using the base R function approx()) each genes’ expression to a standard length of 50 timepoints, covering the full span of ages in BrainSpan, and resolving the sparsity in the BrainSpan matrix. Lastly, for each gene, we z-scaled each timepoint using the base R function scale(), so expression levels are comparable across timepoints. From this processing we derive a total of 800 measures per gene, representing expression levels across 16 brain regions at 50 different timepoints.

The STRING database (version 10) was obtained from the online browser^15^. The format of this data is a matrix of gene-by-gene interaction scores, based on a combination of curated evidence types, such as coexpression, protein interaction, and co-mention in literature. As most genes do not interact in STRING, a gene-by-gene interaction matrix is very sparse, with roughly 98% of gene pairs lacking an interaction score. To decrease the sparsity of this gene-by-gene interaction matrix, we converted these scores into a shortest paths matrix. To accomplish this, we filtered out low-confidence interactions from STRING (defined as a score < 0.4), increasing the downstream diversity of shortest paths values. Next, this interaction matrix was converted into an adjacency matrix, from which a shortest paths matrix was calculated. This processing of the STRING database produced a gene-by-gene matrix where each value represents the minimum number of steps needed to traverse the STRING graph from the source gene to the target gene. Full code for downloading and processing the BrainSpan and STRING data is available in the script 01_load_data.R on forecASD’s github page.

TADA summary statistics were downloaded from the largest meta-analysis for autism available at the time of development^17^. These summary statistics included the most informative column, tadaFdrAscSscExomeSscAgpSmallDel, which integrates data from the SSC and ASC cohorts^3^ across multiple mutation types, including *de novo* loss of function and small deletions. TADA summary statistics were also obtained from the supplementary table of another comprehensive study of autism risk^3^. All five available TADA summary statistics were used as features in the final model, TADA_BF, tadaFdrAscSscExomeSscAgpSmallDel, tadaFdrAscSscExome, tadaFdrAscExome, and tadaFdrSscExome.

### Model training and genome wide prediction

All models were trained using SFARI HC genes as positive examples (of which there are 76 common to both STRING & BrainSpan), and a randomly sampled set of 1,000 background genes (i.e., not listed in the SFARI Gene database) as negative examples.

The first level of our stacked model consists of two genome-wide scores based on data from BrainSpan or the STRING interaction network.

The BrainSpan random forest model was trained to classify each of the 1,076 training set genes (*y*) using the 800 BrainSpan observations (*x*) as features. As described in the BrainSpan data assembly methods section, these 800 features represent expression levels at 50 different timepoint (from 8 weeks post-conception to 40 years old) across 16 different brain regions.

The STRING random forest model was trained to classify the training set genes (*y*) using the gene-by-gene shortest paths matrix (*x*) as features. As described in the STRING data assembly methods section, each gene has a set of 18,884 features representing the shortest path from that gene to all other genes in the STRING network. Given the large number of features for the STRING-based random forest model, we performed a backwards-elimination feature selection. To accomplish this, we first fit a random forest model using the full STRING feature set. Then, each STRING feature not selected in any of the constructed trees was dropped. This variable selection step was repeated until the final model contained only features which were selected at least once during tree construction.

The random forest models were trained with 1000 total trees in a balanced fashion, with 70 positive and 70 negative training examples during the construction of each tree. The randomForest package^18^ (version 4.6-12) in R^16^ (version 3.5.1) was used for all model training. With the trained STRING and BrainSpan models, we then predicted autism involvement scores for the remaining genes not included in our training set. These scores are in Supplementary Table S1 in the columns BrainSpan_score and STRING_score. Reported scores for training set genes are the out-of-bag (OOB) estimates, providing an unbiased estimate of training set genes where they were not used in training of the trees used to make their estimate.

We used these scores (test set gene predictions and OOB training set gene predictions), along with DAWN^7^, TADA^3,17^, DAMAGES^9^, and the score from Krishnan *et al*.^8^, as predictive features in a final ensemble Random Forest, using the same 1,076 training labels described above. Genome-wide predictions were then obtained, again using OOB estimates for training set genes. This final score is listed under the forecASD column in Supplementary Table S1.

### Performance comparisons using *de novo* mutation data and SFARI gene

**Figure 2:**
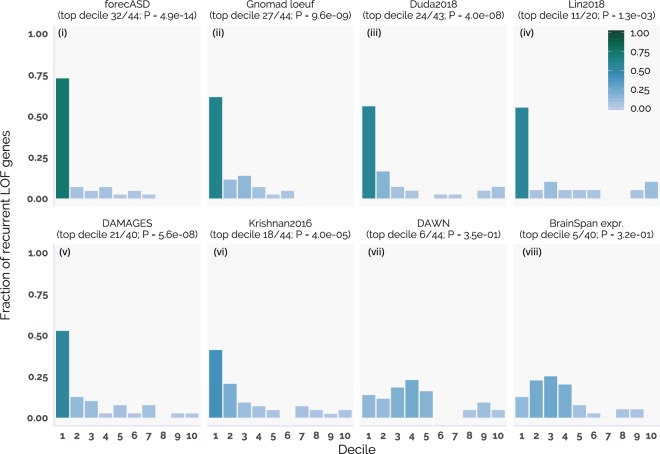
Performance comparison of forecASD with competing methods in prioritizing genes hit by recurrent de novo loss-of-function mutations across three independent ASD cohorts. Bar height and color indicate the fraction of genes with recurrent LOF mutations (n = 44) within the given decile of genes ranked by each score. A binomial test was used to assess for an enrichment of genes with recurrent LOF mutations in the top decile of each score, using each scores decile enrichment for proband synonymous mutations as a baseline (result listed in parentheses below score name).

*De novo* mutation (DNM) data from the MSSNG dataset^12^ was obtained through the denovo-db database^19^. DNM data was also obtained from the SPARK pilot dataset from the consortium’s recently released *de novo* mutation table^20^. Lastly, DNM data was obtained from the recently released Autism Sequencing Consortium (ASC) update^6^, and subset to contain only newly sequenced ASC samples (n = 6,197). Across all three datasets, only DNMs from probands were kept. To compare the performances of the different gene prioritization methods (Fig. 2), a set of 44 genes was found which had at least two or more *de novo* loss-of-function mutations (hereafter referred to as recurrent LOF) in the combined MSSNG, ASC, and SPARK datasets. A binomial test was used to calculate if the first decile of genes prioritized by each method were enriched for these 44 genes. The expected proportion of genes contained within the first decile was calculated for each method separately using the proportion of genes in the first decile containing a synonymous mutation in probands. This adjustment corrects for scores that highly rank genes with high mutation rates. This is a known issue for brain expressed genes, which tend to accumulate mutations at elevated rates due to their above average length^21^.

As an additional performance test, all methods were also evaluated for their ability to prioritize SFARI Gene score 3 genes (Supplementary Fig. 3). Importantly, this test was performed to control for the influence of TADA-based features when prioritizing potential ASD risk genes (which should be enriched in SFARI Gene score 3 genes). To carry out this test, a logistic regression model was fit to predict SFARI Gene score 3 genes, using all other genes not contained in SFARI Gene (any score category) as a negative set. The features in each logistic regression model were one of the competing methods’ score and all five TADA-based measures used by forecASD. The within-sample genome-wide predicted values from all models was used to plot the ROC curve (Supplementary Fig. 3), and the Z-score and p-value of all methods coefficient is reported in the text.

### ASC and iHART novel ASD risk gene data sources

**Figure 3:**
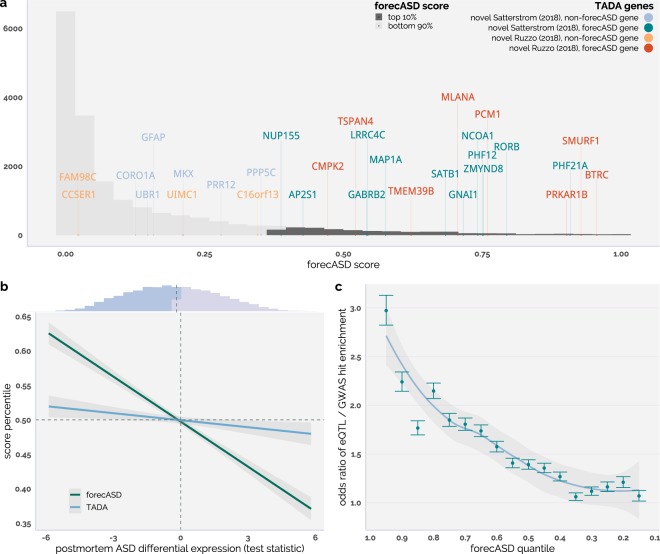
forecASD prioritizes ASD genetic signal across diverse data types. The majority of novel ASD genes recently identified in two ASD sequencing studies by TADA (not part of forecASD’s training set) fall within the top decile of forecASD (**a**). The forecASD score is significantly negatively correlated with ASD postmortem differential expression levels (**b**). Higher scoring forecASD genes are enriched for brain eQTL that also have low p-values (nominal < 0.01) in ASD GWAS (**c**).

Gene lists were taken from the Autism Sequencing Consortium (ASC)^6^ and the Hartwell Autism Research and Technology Initiative (iHART)^22^ preprints. These lists were filtered, excluding genes that were already present in the SFARI gene database^4^, and used in Fig. 3a. The iHART dataset is an ASD cohort with whole genome sequencing on multiplex families (n = 2,308), while the ASC cohort is a collection of trios.

### Pathway enrichment and comparison with case/control brain gene expression data

**Figure 4:**
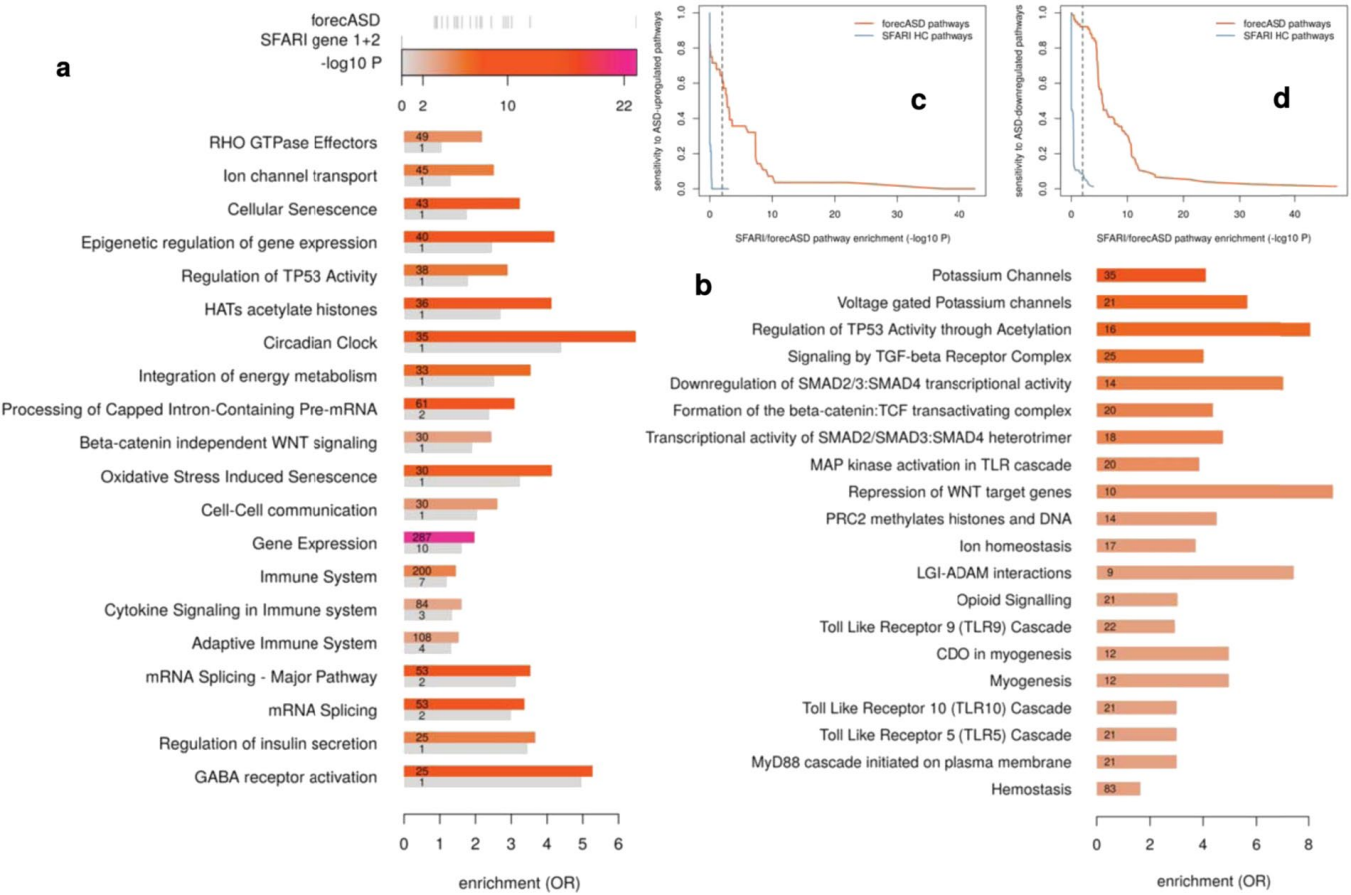
Pathway analysis of genes highlighted by forecASD. When testing the top-decile genes according to forecASD for Reactome pathway enrichment, pathways emerged that were represented, but not enriched in the SFARI HC list (**a**). In panel a, for each pathway, the top bar represents the number of genes (number) and the enrichment (color) for that pathway in top decile forecASD genes, while the bottom represents the enrichment for that pathway in SFARI HC genes. Other pathways were highly enriched in forecASD genes that were not represented at all in the SFARI HC list, even though they have associated literature suggesting a role in autism (**b**). forecASD is more sensitive than SFARI HC to pathways that are differentially regulated in the brains of individuals with autism, particularly in ASD-upregulated pathways (**c**), but also in downregulated pathways (**d**). Using the top decile of TADA (− log10 FDR) genes showed similar sensitivity to SFARI HC (not shown), suggesting that rare variant approaches may be less sensitive in implicating genes found through gene expression studies.

We used Reactome annotations^23^, and unless otherwise noted, PantherDB^24^ to assess functional enrichment in both forecASD genes and SFARI HC genes using Fisher’s method. Odds ratios and p-values were used to compare these two prioritization methods (Fig. 4) in terms of the pathways they implicate. The full list of results of these enrichment analyses are provided in Supplemental Table S2. Statistical analyses described in results and discussion were performed in R^16^ using either glm() or fisher.test(). Gene-wise and pathway-level comparisons with ASD case/control brain gene expression data were performed using frontal cortex RNA-seq summary statistics^25^.

### Brain eQTL analysis

Brain expression quantitative trait loci (eQTL) passing a Bonferroni FDR significance level of 0.05 and filtered for genes having expression >0.1 FPKM in at least 150 samples from PsychENCODE^26^ were selected for further analysis. In parallel, these eQTL were matched to SNPs in a recent autism GWAS^27^. eQTL that did not map to a SNP from the ASD GWAS were excluded from further analysis. eQTL were then split into two classes, based on whether their associated ASD GWAS match had an unadjusted p-value above or below 0.01. eQTL target genes were then split into 5% quantiles by their associated genes’ forecASD score. A Fisher’s test was performed comparing the top 5% quantile to all lower bins, testing for an enrichment of eQTL with ASD GWAS p-values below 0.01 (termed eQTL/GWAS hit enrichment). To visualize this test (Fig. 3c), the odds ratio for eQTL/GWAS hits for each bin (and all preceding bins) relative to all lower bins is shown.

### Cluster analysis of top scoring forecASD genes

**Figure 5:**
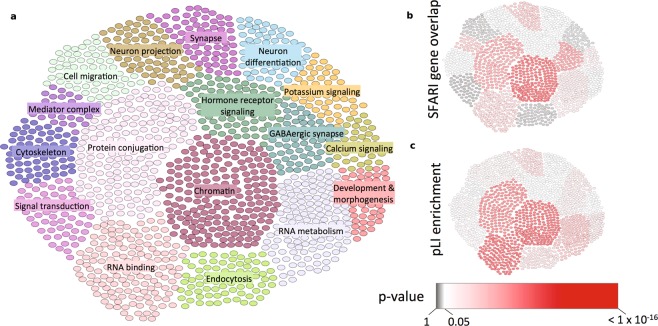
Clustering of genes highlighted by forecASD into distinct modules. Greedy hierarchical optimization of the modularity score yielded 17 clusters consisting of 1,452 forecASD genes (**a**). All clusters have several significantly enriched biological pathways, of which the top terms were labeled. Clusters were tested for significance of overlap with the list of SFARI HC genes (**b**), and enrichment of haploinsufficiency genes (pLI > 0.5; **c**).

Using the STRING database, interactions were obtained for forecASD genes (top decile) and used to create a network with the igraph package^28^ in R^16^. No filter was applied to the interaction score. Hierarchical greedy clustering based on optimization of modularity score^29^ was performed using the fastgreedy.community() function from the igraph package^28^. Clustering was performed iteratively, with clusters containing more than 200 genes subject to further clustering, and clusters with fewer than 30 genes discarded. The annotated network of clusters was loaded into Cytoscape^30^, using the STRING application^31^. Functional enrichment of clusters was assessed using the STRING application in Cytoscape, with the p-value threshold set to 0.05. For annotation of the network plot shown in Fig. 5a, either the top annotated term or consensus among several top terms was chosen as the representative label. The enrichment of pLI was assessed using Fisher’s exact test on genes within each cluster with a pLI score above 0.5. The assessment of enrichment with SFARI HC genes was performed using the hypergeometric test, assuming a background of 18,000 total genes (the number of genes to which a forecASD score could be assigned).

### Ethics approval and consent to participate

All human genetic data used in this study was accessed in a deidentified manner from associated sequencing consortia with their approval. Due to the method of access, this study is not formally considered human subjects research and therefore not subject to associated restrictions, as outlined by the National Institutes of Health.

## Results

### forecASD model performance

We first tested forecASD’s ability to identify putative risk genes by assessing enrichment of *de novo* mutations in genes observed in three recent data sets not used in forecASD’s development or training: the SPARK pilot^13,20^, MSSNG^12^, and the 2018 release of ASC samples^6^. A robustly reproducible result from ASD sequencing studies is an enrichment of *de novo* LOF mutations in autism-associated genes in probands^32–34^. Combining all *de novo* mutations across the three cohorts into a single table, we used a binomial test (Fig. 1b) to examine whether genes affected by each mutation class (recurrent LOF, singleton LOF, recurrent missense) showed an over-representation in the top decile of forecASD scores. The expected proportion of genes (0.196) within the top decile was calculated using the percentage of genes with *de novo* synonymous mutations in the top decile across the three cohorts (Fig. 1b.i.). From this test, we found a significant enrichment (Fig. 1b.ii.) of recurrent LOF mutations (observed in more than one individual) in the top decile of forecASD genes (binomial test of first decile, P = 4.9 × 10^−14^). This same binomial test was performed for singleton LOF and recurrent missense *de novo* mutations (Fig. 1b.iii–iv.), which also showed a significant enrichment in the top decile (P = 3.4 × 10^−6^ and 3.5 × 10^−5^, respectively). Because substantial enrichment of LOF mutations was found in the top decile (n = 1,787 genes), we use use this set of genes (“forecASD genes”) in subsequent analyses.

To evaluate forecASD’s performance relative to other methods, we extended the above decile enrichment tests of recurrent *de novo* LOF genes to several other gene prioritization methods (Fig. 2). For non-autism specific baseline scores, we used the recently released gnomad LOEUF score (Fig. 2.ii.)^35^ (which quantifies the depletion of LOF mutations in genes across a large population sample), and total expression within the BrainSpan dataset (Fig. 2.viii.)^14^ (which prioritizes genes highly expressed across numerous brain regions). Alternative autism risk gene prioritization methods include Duda *et al*.^11^, Lin *et al*.^10^, DAMAGES^9^, Krishnan *et al*.^8^ and DAWN^7^. Across all scores tested, forecASD showed the greatest enrichment for recurrent *de novo* LOF mutations in its top decile (binomial test, 32/44 genes, P = 4.9 × 10^−14^). After forecASD, loeuf showed the second greatest enrichment (27/44, P = 9.6 × 10^−9^), followed by Duda^11^ (24/43, P = 3.9 × 10^−8^), Lin^10^ (11/20, P = 1.2 × 10^−3^), DAMAGES^9^ (21/40, P = 5.6 × 10^−8^), Krishnan^8^ (18/44, P = 4.0 × 10^−5^), DAWN^7^ (6/44, P = 3.9 × 10^−1^), and lastly BrainSpan expression (5/40, P = 3.2 × 10^−1^). Importantly, MSSNG, SPARK, and ASC data were not used directly or indirectly in the training of the forecASD ensemble (e.g., SFARI gene training labels retrieved before SPARK, MSSNG, and recent ASC release, TADA features also prior to release of 3 cohorts), and therefore demonstrate the generalization of these methods to unseen data.

As a further performance benchmark, we tested the predictive strength of all evaluated models when controlling for TADA scores. Since TADA scores are used to define new ASD risk genes, most gene prioritization methods, including forecASD, are either directly or indirectly using TADA scores. To examine signal outside of TADA, we fit logistic regression models including all five TADA-based features used by forecASD as covariates (see Methods section, BrainSpan, STRING, and TADA data assembly), with each of the methods scores as the predictor of membership among SFARI Gene score 3 genes (Supplemental Fig. S3; score 3 genes were not used in training forecASD, but are nevertheless assumed to be enriched for true ASD genes). All other genes listed in the SFARI Gene database were removed from the analysis, and the remainder of the genome was considered the negative class. Strong association with score 3 membership (as measured by the regression coefficient and Z-score) is a desirable trait for an ASD gene prediction method, and forecASD proved to be the most strongly associated of the tested methods, even after correcting for the information imparted by TADA rankings (Z = 13.35, P = 1.19 × 10^−40^). Following forecASD was Lin (Z = 11.2, P = 3.65 × 10^−29^), Duda (Z = 8.56, P = 1.09 × 10^−17^), LOEUF (Z = −8.52, P = 1.50 × 10^−17^), Krishnan (Z = 8.49, P = 1.99 × 10^−17^), DAMAGES (Z = 4.82, P = 1.38 × 10^−6^), DAWN (Z = 3.92, P = 8.68 × 10^−5^), and BrainSpan (Z =  − 0.4, P = 6.6 × 10^−1^).

### Applications of forecASD: risk gene discovery, differential expression, GWAS

A primary goal of genetic research of ASD is enumerating the full complement of genes that confer risk when disrupted. A central claim of this work is that forecASD scores “anticipate” which genes will eventually show evidence of association in gene discovery studies. Toward this end, we tested the enrichment of high-scoring forecASD genes among the findings of two large, recent ASD sequencing studies, from the ASC^6^ and the iHART^22^ consortia. These studies were published after the development and training of forecASD. From this analysis (Fig. 3a) we found that 12/18 and 8/12 novel candidate genes reported by the ASC and iHART consortia, respectively, were top-decile forecASD genes, and were significantly enrichmented for high forecASD scores (Wilcoxon rank sum test; P = 4.98 × 10^−8^; P = 8.87 × 10^−14^). However, as is common practice in ASD genetic studies, both the ASC and iHART studies include previously published cohorts, such as the Simons Simplex Collection, in their gene discovery efforts. As genetic information from these cohorts was used in the training of forecASD (i.e. TADA-based features), this raises the question if forecASD’s signal is purely driven by cohort overlap. To test for this possibility, we fit a logistic regression model predicting whether a gene was one of the novel candidate genes (fit across all 17,881 non-SFARI HC genes, with 30 positive class examples) using forecASD score and all genetic features (i.e. all 5 TADA-based features) as predictors. A Wald test showed that forecASD significantly improved prediction (P = 1.73 × 10^−9^) beyond a baseline model including all genetic features. These analyses support the observation that forecASD generalizes well and is a good indicator of actual genetic risk for autism.

Extending the forecASD score to postmortem brain gene expression studies, we accessed the largest such study available to date^25^, which includes 51 ASD postmortem brain samples. In general, relatively little overlap has been observed between differential expression findings in ASD postmortem brains and genes implicated by genetic association studies. For example, in the study referenced^25^, only 2/76 genes scoring either 1 or 2 in SFARI gene were found in the set of 1824 differentially expressed genes (all brain regions combined). Despite this low prior expectation, we found that high-scoring forecASD genes are enriched among downregulated genes (Fig. 3b) from ASD postmortem case/control gene expression studies (OR = 1.3, P = 0.0013), and are depleted from upregulated genes (OR = 0.57, P = 1.8 × 10^−8^). This forecASD-differential expression signature is more significant than a comparable analysis using TADA scores to prioritize genes (P < 2.2 × 10^−16^), suggesting forecASD is more sensitive to ASD-associated genes discovered through differential expression studies.

To determine forecASD’s utility in identifying a role for common variation in ASD risk, we analyzed the connection between brain eQTL and ASD GWAS signatures. Specifically, we integrated a recently published list of brain eQTL^26^ with a recent ASD GWAS study^27^. From this analysis (Fig. 3c), we found that brain eQTL for forecASD genes (top decile) were more likely to be nominally ASD-associated SNPs (binary cutoff of P < 0.01) than brain eQTL for the bottom 90% of genes scored by forecASD (Fisher’s test: odds ratio = 2.24, P = 4.89 × 10^−244^).

### Pathway and network analysis of forecASD genes

After demonstrating the predictive performance characteristics of forecASD, we turned to practical applications that could further illuminate the underlying biological mechanisms at play in autism. Functional enrichment using Reactome annotations showed forecASD genes are highly enriched for pathways known to play important roles in autism etiology, including chromatin modification, synaptic transmission, and developmental biology (full list in Supplemental Table S2). To highlight new biological themes that forecASD detects — but that are not clear from the list of SFARI HC genes — we prioritized pathways based on differential enrichment (Fig. 4). Figure 4a highlights pathways represented in SFARI HC genes, but that showed significantly greater enrichment in forecASD genes. Figure 4b shows a sampling of the forecASD pathways with the most significant p-values that are not represented by any SFARI HC gene, highlighting under-appreciated mechanisms in autism.

Given the existing gap between ASD postmortem differential expression findings and known ASD risk genes, we also analyzed pathway enrichment overlaps between either SFARI HC or forecASD genes with postmortem differentially expressed genes. We found forecASD has increased sensitivity to autism risk pathways identified in gene expression studies, for both ASD upregulated (Fig. 4c) and downregulated pathways (Fig. 4d). Overall, pathways demonstrating forecASD-specific excess enrichment showed a significant agreement with pathway enrichment from independent case/control brain gene expression studies (OR = 28.8; P = 2.9 × 10^−48^), and were more likely to support pathways that were up-regulated in the gene expression data (OR = 2.1, P = 1.27 × 10^−6^, Fig. 4c) compared to pathways implicated by SFARI HC.

For a global view of forecASD gene function, we performed a network analysis using forecASD top decile genes. The resulting network consisted of 17 clusters composed of 1,452 genes (Fig. 5a). All clusters were significantly enriched with numerous GO and KEGG pathways (Supplemental Table S3). Clusters were also tested for overlap with SFARI HC genes by a hypergeometric test, and 8 clusters were not significantly enriched for these genes, suggesting forecASD identified groupings of genes currently missing from the known list of autism risk genes (Fig. 5b). The clusters lacking significant overlap included those with the functions: signal transduction, cytoskeleton, cell migration, neuron projection, steroid signaling, neuron differentiation, potassium signaling, development and morphogenesis. All clusters — except for a small cluster with functions related to mediator complex — were significantly enriched for haploinsufficient genes (pLI > 0.5) (Fig. 5c) by a Fisher’s test. Similar to the pathway analysis above, we found an entire cluster enriched for potassium signaling genes (P = 1.8 × 10^−49^) which lacked significant overlap with SFARI HC genes. Other notable examples include clusters related to cell migration (P = 6.0 × 10^−11^) and endocytosis (P = 2.7 × 10^−21^). These pathways have recently been explored for their ability to regulate brain connectivity^36^ and postsynaptic organization^37^, respectively.

## Discussion

We present forecASD, a machine learning approach that combines systems biology and genetic models into a single score that indexes the strength of evidence for a gene’s involvement in autism. This genome-wide score can be a useful prior, filter, or positive control in molecular studies of autism. It can also be used as a starting point to generate new hypotheses to investigate currently under-appreciated aspects of the molecular etiology of autism. In our tests of predictive performance and generalization, forecASD outperformed other systems biology and genetic approaches for autism risk gene prioritization.

Applying forecASD to two recent, sequencing-based ASD risk gene discovery efforts^6,22^ showed that forecASD ranked their proposed novel candidate risk genes highly (Fig. 3a). We foresee this as a central application of forecASD, allowing candidate risk genes to be prioritized based on a diversity of ASD-associated signal. Going further, we applied forecASD to the differential expression patterns seen in postmortem ASD brain samples. The lack of overlap between ASD disease genes and gene expression based studies has been speculated to be due to differential expression results capturing downstream effects of ASD, while sequencing results are capturing upstream, causative effects^25^. We found that high-scoring forecASD genes were more likely to be downregulated, and conversely, high-scoring forecASD genes were less likely to be upregulated (Fig. 3b). This is the first report to our knowledge of global downregulation of ASD risk genes in ASD postmortem brains. Though previously unreported, this pattern is in line with expectations, as ASD risk genes are largely found through an accumulation of gene-disrupting mutations. Lastly, we applied the forecASD score in an analysis of common risk variants to prioritize brain eQTL (Fig. 3c), finding that eQTL associated with high-scoring forecASD genes showed a significant enrichment for ASD GWAS signal. In addition to serving as a further validation of forecASD’s gene prioritization, this demonstrates how forecASD can be used to prioritize common, intergenic variation in order to find enrichment of ASD-specific signal.

Beyond genes, the organization of ASD risk into distinct biological pathways has also been central to our understanding of the condition’s pathology. Some pathways, although represented in the current SFARI HC list, showed a substantial relative increase in enrichment when considering forecASD (Fig. 4a, Supplemental Table S2). These pathways may therefore represent noted and plausible — but still under-appreciated — molecular themes in our understanding of autism. The pathway with the largest relative increase in enrichment from SFARI HC to forecASD was Rho GTPase signaling (OR = 2.2, P = 4.8 × 10^−5^), which plays a critical role in cytoskeletal dynamics in neurodevelopment^38^, including interactions with SHANK proteins and the formation and maturation of dendritic spines^39^. As another example, although chromatin modification is a well-established theme in autism genetic risk, histone acetyltransferases showed relatively little representation in the SFARI HC list, but were significantly enriched in forecASD genes (OR = 4.1, P = 3.5 × 10^−9^). Histone acetylation was recently shown to be a pervasive genomic predictor of affected status in a large autism case/control postmortem brain study^40^. It appears that this mechanism’s importance was under-represented by established risk genes, but forecASD is sensitive to it.

As a final example of under-appreciated molecular mechanisms, the disruption of circadian clock pathway genes was implicated by forecASD as an important source of risk for autism (OR = 6.5, P = 7.6 × 10^−13^). Sleep disturbances are a well-known and problematic comorbidity in autism, and molecular deficits in circadian regulation related to autism have been documented^41–43^. Although literature support is available for these processes playing a role in autism, our results indicate that their current sparse representation in lists of accepted genetic risk factors is not representative of their importance in the disorder.

Other pathways were identified by forecASD as significantly enriched for autism risk, but were not represented at all among SFARI HC genes (Supplemental Table S2, Fig. 4b). Consequently, we expect these pathways to contribute to new insights into the molecular basis of autism. Potassium channels showed particularly significant enrichment in forecASD genes (OR = 4.1, P = 7.2 × 10^−9^, N = 35 genes) despite the absence of potassium channel genes among currently accepted autism risk genes. However, literature shows support for a role for potassium channels in ASD risk^44–47^, and the pathway was enriched for differential regulation in a recently published brain gene expression study of autism (P = 0.001, downregulated)^25^. Overall, when comparing the pathways identified using forecASD genes, we found a significant agreement with pathways enriched in an ASD case/control postmortem brain gene expression study^25^ (Fig. 4). Notably, the agreement between forecASD and ASD postmortem brain up-regulated pathways was significantly stronger than the agreement between SFARI HC and postmortem brain upregulated pathways. This finding highlights a potential blindspot of current ASD gene discovery methods to pathways which have experimental evidence of involvement in ASD.

Using TADA to impart genetic association information to the forecASD ensemble is unique among the ASD gene prediction approaches we used as benchmarks. However, this raises concerns about the potential for circularity: TADA is emerging as the most popular way to compute and update gene-wise genetic association statistics for ASD studies, and previous TADA scores are strongly correlated with updated TADA scores. Furthermore, TADA scores are among the most important predictive features in the forecASD ensemble (Supplementary Fig. S2). Consequently, it would be concerning if forecASD’s ability to predict new ASD genes was due entirely to the inclusion of previously published TADA scores. Testing for this possibility, we found that forecASD remained the strongest predictor of SFARI score 3 genes (not used during training), even when using all five TADA-based features as covariates in a logistic regression model (Supplemental Fig. S3).

Although our cluster analysis identified well-defined clusters with an enrichment for haploinsufficient genes, we observed only a marginally significant relationship between cluster pLI enrichment and SFARI HC gene overlap. The lack of signal was likely driven by the relatively even distribution of SFARI HC genes among clusters. This suggests that — across the functionally coherent groups of genes forecASD identified — there is no bias in existing knowledge toward one cluster or another, and that these clusters may represent yet-undiscovered reservoirs of ASD risk genes. We believe this means that forecASD can help point the way forward to as-yet undiscovered ASD biology.

For the foreseeable future, traditional gene discovery studies will continue to add to the list of bona fide ASD risk genes. When sample sizes saturate and gene discovery decelerates, the field will be challenged to develop new and useful applications of this acquired knowledge. By combining new and previously published predictors into a high-performance ensemble classifier, forecASD provides a glimpse of this future and gives an opportunity to begin thinking about what can be done with a comprehensive list of autism risk genes.

## Supplementary information

Supplementary Information.

## Data Availability

Approved researchers can obtain the SPARK population dataset described in this study by applying at https://base.sfari.org. All data and code used to generate the forecASD model is available for download from the repository associated with this project, https://github.com/LeoBman/forecASD.

## References

[1] Rosenberg RE (2009). Characteristics and concordance of autism spectrum disorders among 277 twin pairs. Archives of Pediatrics & Adolescent Medicine.

[2] Colvert E (2015). Heritability of autism spectrum disorder in a UK population-based twin sample. JAMA Psychiatry.

[3] Rubeis SD (2014). Synaptic, transcriptional and chromatin genes disrupted in autism. Nature.

[4] Abrahams BS (2013). SFARI gene 2.0: a community-driven knowledgebase for the autism spectrum disorders (ASDs). Molecular Autism.

[5] Iossifov I (2014). The contribution of de novo coding mutations to autism spectrum disorder. Nature.

[6] Satterstrom, F. K. *et al*. Large-scale exome sequencing study implicates both developmental and functional changes in the neurobiology of autism 10.1101/484113 (2018). 10.1016/j.cell.2019.12.036 PMC7250485 31981491

[7] Liu L (2014). DAWN: a framework to identify autism genes and subnetworks using gene expression and genetics. Molecular Autism.

[8] Krishnan A (2016). Genome-wide prediction and functional characterization of the genetic basis of autism spectrum disorder. Nature Neuroscience.

[9] Zhang C, Shen Y (2016). A cell type-specific expression signature predicts haploinsufficient autism-susceptibility genes. Human Mutation.

[10] Lin, Y., Rajadhyaksha, A. M., Potash, J. B. & Han, S. A machine learning approach to predicting autism risk genes: Validation of known genes and discovery of new candidates 10.1101/463547 (2018). 10.3389/fgene.2020.500064 PMC7513695 33133139

[11] Duda, M. *et al*. Brain-specific functional relationship networks inform autism spectrum disorder gene prediction. *Translational Psychiatry* **8**10.1038/s41398-018-0098-6 (2018). 10.1038/s41398-018-0098-6 PMC5838237 29507298

[12] Yuen RKC (2017). Whole genome sequencing resource identifies 18 new candidate genes for autism spectrum disorder. Nature Neuroscience.

[13] Feliciano P (2018). SPARK: A US cohort of 50, 000 families to accelerate autism research. Neuron.

[14] Sunkin SM (2012). Allen brain atlas: an integrated spatio-temporal portal for exploring the central nervous system. Nucleic Acids Research.

[15] v. Mering C (2003). STRING: a database of predicted functional associations between proteins. Nucleic Acids Research.

[16] R Development Core Team. *R: A Language and Environment for Statistical Computing*. R Foundation for Statistical Computing, Vienna, Austria (2008). ISBN 3-900051-07-0.

[17] Sanders SJ (2015). Insights into autism spectrum disorder genomic architecture and biology from 71 risk loci. Neuron.

[18] Liaw A, Wiener M (2002). Classification and regression by randomforest. R News.

[19] denovo-db.gs.washington.edu. Accessed: 2018.

[20] Feliciano, P. *et al*. Exome sequencing of 457 autism families recruited online provides evidence for novel asd genes. *bioRxiv*, 10.1101/516625, https://www.biorxiv.org/content/early/2019/44101/09/516625.full.pdf (2019). 10.1038/s41525-019-0093-8 PMC6707204 31452935

[21] Zylka MJ, Simon JM, Philpot BD (2015). Gene length matters in neurons. Neuron.

[22] Ruzzo, E. K. *et al*. Whole genome sequencing in multiplex families reveals novel inherited and de novo genetic risk in autism, 10.1101/338855 (2018).

[23] Fabregat A (2017). The reactome pathway knowledgebase. Nucleic Acids Research.

[24] Mi H (2004). The PANTHER database of protein families, subfamilies, functions and pathways. Nucleic Acids Research.

[25] Gandal MJ (2018). Shared molecular neuropathology across major psychiatric disorders parallels polygenic overlap. Science.

[26] Wang D (2018). Comprehensive functional genomic resource and integrative model for the human brain. Science.

[27] Grove J (2019). Identification of common genetic risk variants for autism spectrum disorder. Nature Genetics.

[28] Csardi, G. & Nepusz, T. The igraph software package for complex network research. *InterJournal* **Complex Systems**, 1695 (2006).

[29] Newman MEJ (2006). Modularity and community structure in networks. Proceedings of the National Academy of Sciences.

[30] Shannon P (2003). Cytoscape: A software environment for integrated models of biomolecular interaction networks. Genome Research.

[31] Szklarczyk D (2016). The STRING database in 2017: quality-controlled protein-protein association networks, made broadly accessible. Nucleic Acids Research.

[32] O’Roak BJ (2012). Sporadic autism exomes reveal a highly interconnected protein network of de novo mutations. Nature.

[33] Sanders SJ (2012). De novo mutations revealed by whole-exome sequencing are strongly associated with autism. Nature.

[34] Turner TN (2017). Genomic patterns of de novo mutation in simplex autism. Cell.

[35] Karczewski, K. J. *et al*. Variation across 141, 456 human exomes and genomes reveals the spectrum of loss-of-function intolerance across human protein-coding genes 10.1101/531210 (2019).

[36] Reiner O, Karzbrun E, Kshirsagar A, Kaibuchi K (2015). Regulation of neuronal migration, an emerging topic in autism spectrum disorders. Journal of Neurochemistry.

[37] Loebrich S (2014). The role of f-actin in modulating clathrin-mediated endocytosis: Lessons from neurons in health and neuropsychiatric disorder. Communicative &Integrative Biology.

[38] Reichova A, Zatkova M, Bacova Z, Bakos J (2017). Abnormalities in interactions of rho GTPases with scaffolding proteins contribute to neurodevelopmental disorders. Journal of Neuroscience Research.

[39] Martin-Vilchez S (2017). RhoGTPase regulators orchestrate distinct stages of synaptic development. PLOS ONE.

[40] Sun W (2016). Histone acetylome-wide association study of autism spectrum disorder. Cell.

[41] Lipton JO (2017). Aberrant proteostasis of BMAL1 underlies circadian abnormalities in a paradigmatic mTOR-opathy. Cell Reports.

[42] Monyak RE (2016). Insulin signaling misregulation underlies circadian and cognitive deficits in a drosophila fragile x model. Molecular Psychiatry.

[43] Kozlov SV (2007). The imprinted gene magel2 regulates normal circadian output. Nature Genetics.

[44] Guglielmi, L. Update on the implication of potassium channels in autism: K channelautism spectrum disorder. *Frontiers Cellular Neuroscience* **9**, 10.3389/fncel.2015.00034 (2015). 10.3389/fncel.2015.00034 PMC4345917 25784856

[45] Deng P-Y, Klyachko VA (2015). Genetic upregulation of BK channel activity normalizes multiple synaptic and circuit defects in a mouse model of fragile x syndrome. The Journal of Physiology.

[46] Lee H (2014). Exome sequencing identifies de novo gain of function missense mutation in KCND2 in identical twins with autism and seizures that slows potassium channel inactivation. Human Molecular Genetics.

[47] Sicca, F. *et al*. Gain-of-function defects of astrocytic kir4.1 channels in children with autism spectrum disorders and epilepsy. *Scientific Reports* **6**, 10.1038/srep34325 (2016). 10.1038/srep34325 PMC5039625 27677466

