# Author Correction: Forecasting risk gene discovery in autism with machine learning and genome-scale data

**DOI:** 10.1038/s41598-020-77832-2

**Published:** 2020-11-26

**Authors:** Leo Brueggeman, Tanner Koomar, Jacob J. Michaelson

**Affiliations:** 1 Department of Psychiatry, University of Iowa, Iowa City, IA USA; 2 Interdisciplinary Genetics Program, University of Iowa, Iowa City, IA USA; 3 Medical Scientist Training Program, University of Iowa, Iowa City, IA USA

Correction to: *Scientific Reports* 10.1038/s41598-020-61288-5, published online 12 March 2020

This Article contains an error in the Supplementary Information, where Supplementary Tables S1–S3 were omitted. These files are provided below.

## Supplementary information

Supplementary Table S1.

Supplementary Table S2.

Supplementary Table S3.

