# Supplementary material for: Forecasting risk gene discovery in autism with machine learning and genome-scale data
Source: Sci Rep. 2020 Mar 12;10:4569. doi: 10.1038/s41598-020-61288-5 (PMC7067874; doi:10.1038/s41598-020-61288-5)
Supplement: Supplementary file 1 — Supplementary Information. [file 41598_2020_61288_MOESM1_ESM.pdf]

Forecasting risk gene discovery in autism with machine learning and genome-scale data

Leo Brueggeman[1,2,3], Tanner Koomar[1,2], Jacob Michaelson[1,2,*]

1: University of Iowa, Department of Psychiatry, Iowa City IA
2: University of Iowa, Interdisciplinary Genetics Program, Iowa City IA
3: University of Iowa, Medical Scientist Training Program, Iowa City IA
Contact information: jacob-michaelson@uiowa.edu

Department of Psychiatry,
University of Iowa, Iowa City IA, USA
11-26-2019

Supplementary materials: forecASD

Leo Brueggeman, Tanner Koomar, Jacob Michaelson

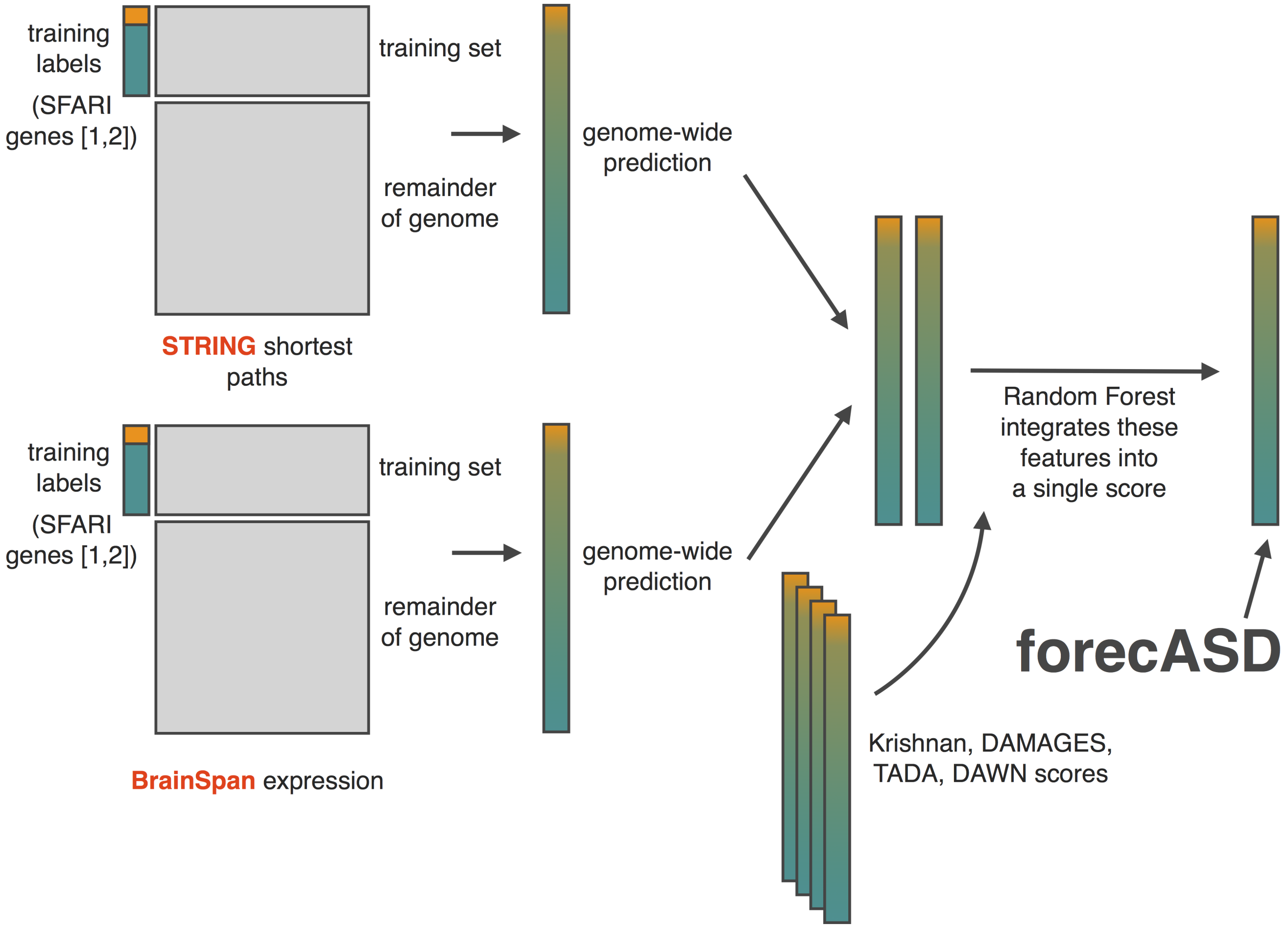

Figure 1. Overview of forecASD model. Two Random Forest classifiers, one using BrainSpan gene expression and the other using the STRING network as predictors, are trained to discriminate high confidence autism genes (SFARI HC, scores 1 and 2) from a set of 1,000 genes drawn randomly from those not listed at all in the SFARI Gene database. Predictions are then made on the remainder of the genome, and these are combined with the out-of-bag (OOB) estimates from the training process to yield a prediction for each gene in the genome. A subsequent classifier is then trained using the output of these two RFs and previously published autism gene scores as predictive features, and again predictions are made on the remainder of the genome, with OOB predictions being used for those genes in the training set. The RF vote proportion for class "autism gene" is then the final forecASD score.

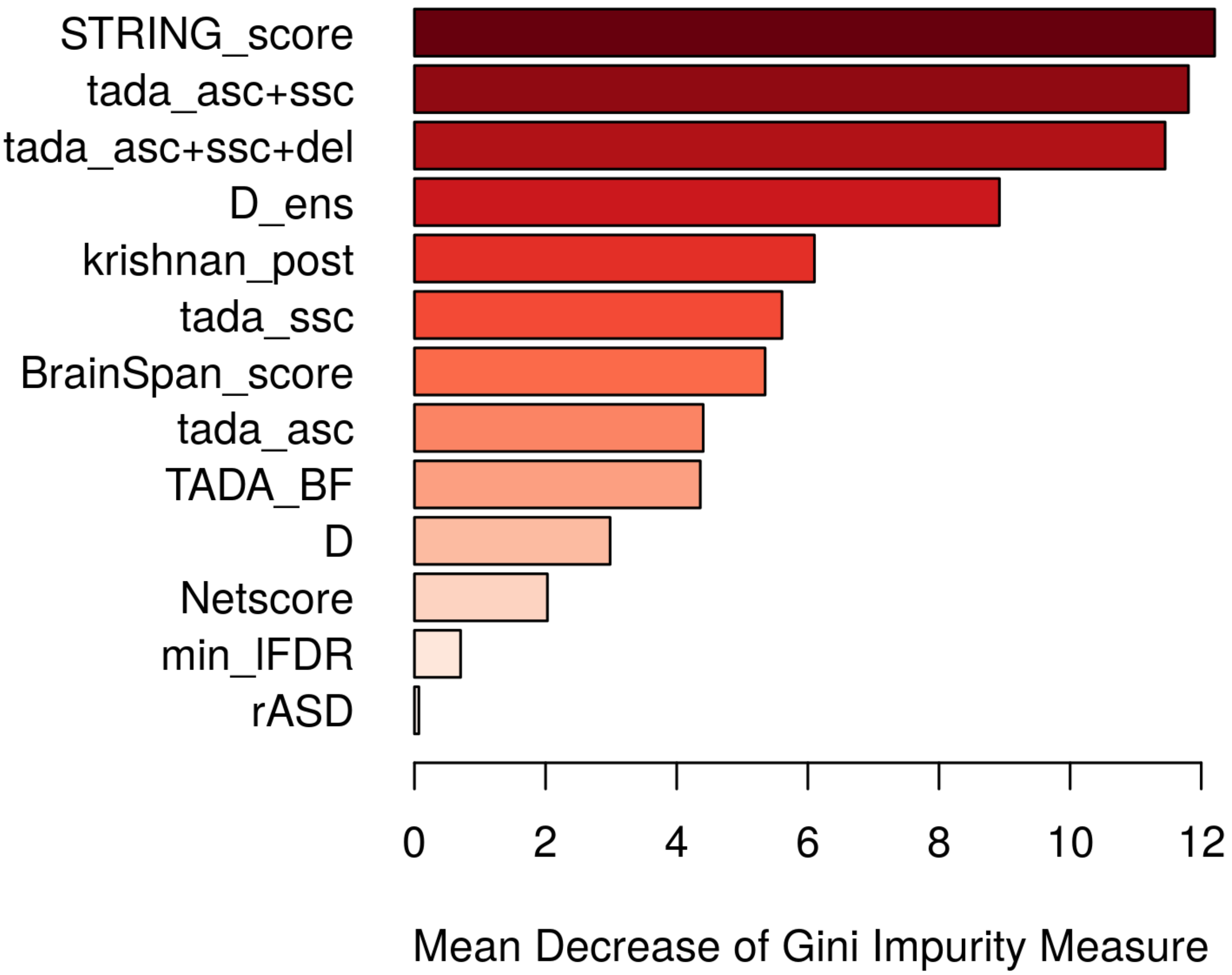

Figure 2. Mean decrease of Gini impurity measure feature importance scores within the forecASD ensemble model are shown, with the STRING score as the single most important feature. STRING score and BrainSpan score are the result of the random forests we trained on SFARI HC genes, in the first layer of the random forest ensemble. Also shown are several TADA summaries from Sanders et al[1] (tada asc+ssc = tadaFdrAscSscExome, tada asc+ssc+del = tadaFdrAscSscExomeSscAgpSmallDel, tada ssc = tadaFdrSscExome, tada asc = tadaFdrAscExome) named after the data sources they use (ASC, SSC cohorts, with/without deletions), and a single, earlier TADA statistic from De Rubeis et al.[2] (TADA BF). Several statistics were taken from DAWN[3], including Netscore, min IFDR and rASD, representing a network-based score, a module based score, and a binary risk gene ASD status score. Lastly, several scores were taken from other ASD gene scoring methods, including D ens and D from DAMAGES[4] and krishnan post from Krishnan et al[5].

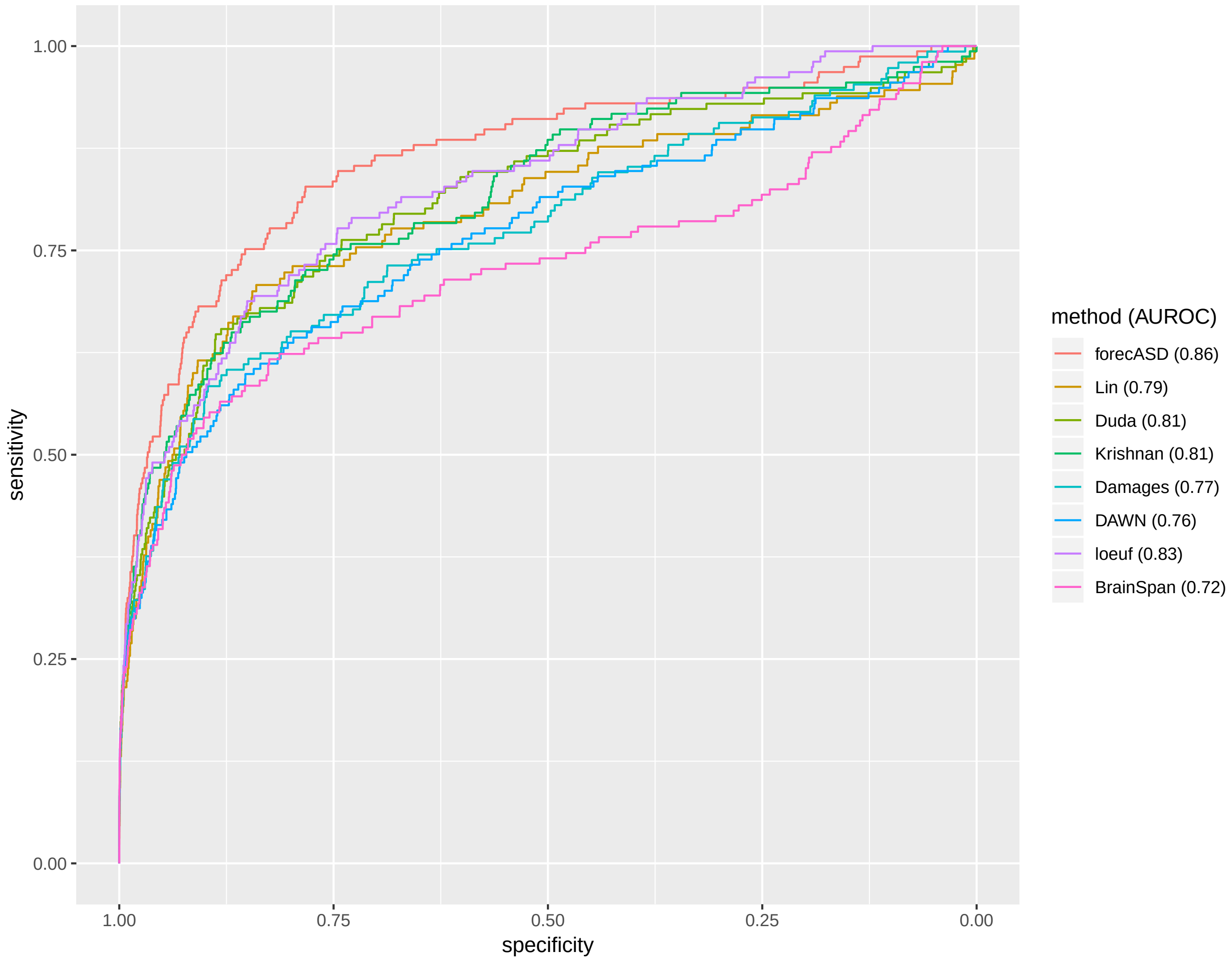

Figure 3. Performance comparison between forecASD and competing methods. Competing methods include Krishnan et al[5], DAMAGES[4], Duda et al[6], DAWN[3], Lin et al[7] and the baseline measures of genes ranked by Gnomad loeuf[8] and BrainSpan gene expression level[9]. All models were fit as part of bivariate logistic regression models, where five TADA based scores were used as covariates to predict SFARI score 3 genes. Area under the ROC curve (AUROC) is shown for each method in parentheses in the legend.

References

1. Sanders, S. J. *et al.* Insights into autism spectrum disorder genomic architecture and biology from 71 risk loci. *Neuron* **87**, 1215–1233, DOI: 10.1016/j.neuron.2015.09.016 (2015).

2. Rubeis, S. D. *et al.* Synaptic, transcriptional and chromatin genes disrupted in autism. *Nature* **515**, 209–215, DOI: 10.1038/nature13772 (2014).

3. Liu, L. *et al.* DAWN: a framework to identify autism genes and subnetworks using gene expression and genetics. *Molecular Autism* **5**, 22, DOI: 10.1186/2040-2392-5-22 (2014).

4. Zhang, C. & Shen, Y. A cell type-specific expression signature predicts haploinsufficient autism-susceptibility genes. *Human Mutation* **38**, 204–215, DOI: 10.1002/humu.23147 (2016).

5. Krishnan, A. *et al.* Genome-wide prediction and functional characterization of the genetic basis of autism spectrum disorder. *Nature Neuroscience* **19**, 1454–1462, DOI: 10.1038/nn.4353 (2016).

6. Duda, M. *et al.* Brain-specific functional relationship networks inform autism spectrum disorder gene prediction. *Translational Psychiatry* **8**, DOI: 10.1038/s41398-018-0098-6 (2018).

7. Lin, Y., Rajadhyaksha, A. M., Potash, J. B. & Han, S. A machine learning approach to predicting autism risk genes: Validation of known genes and discovery of new candidates. DOI: 10.1101/463547 (2018).

8. Karczewski, K. J. *et al.* Variation across 141, 456 human exomes and genomes reveals the spectrum of loss-of-function intolerance across human protein-coding genes. DOI: 10.1101/531210 (2019).

9. Sunkin, S. M. *et al.* Allen brain atlas: an integrated spatio-temporal portal for exploring the central nervous system. *Nucleic Acids Research* **41**, D996–D1008, DOI: 10.1093/nar/gks1042 (2012).
